# Preschoolers’ Development of Theory of Mind: The Contribution of Understanding Psychological Causality in Stories

**DOI:** 10.3389/fpsyg.2018.00955

**Published:** 2018-06-12

**Authors:** Wakako Sanefuji, Etsuko Haryu

**Affiliations:** ^1^Faculty of Human-Environment Studies, Kyushu University, Fukuoka, Japan; ^2^Faculty of Education, The University of Tokyo, Tokyo, Japan

**Keywords:** causality, false belief, theory of mind, preschoolers, development

## Abstract

This study investigated the relationship between children’s abilities to understand causal sequences and another’s false belief. In Experiment 1, we tested 3-, 4-, 5-, and 6-year-old children (*n* = 28, 28, 27, and 27, respectively) using false belief and picture sequencing tasks involving mechanical, behavioral, and psychological causality. Understanding causal sequences in mechanical, behavioral, and psychological stories was related to understanding other’s false beliefs. In Experiment 2, children who failed the initial false belief task (*n* = 50) were reassessed 5 months later. High scorers in the sequencing of the psychological stories in Experiment 1 were more likely to pass the standard false belief task than were the low scorers. Conversely, understanding causal sequences in the mechanical and behavioral stories in Experiment 1 did not predict passing the false belief task in Experiment 2. Thus, children may understand psychological causality before they are able to use it to understand false beliefs.

## Introduction

Imagine that a little girl dawdled in a forest on her way to see her grandmother, and told a wolf where she was going. The wolf took a shortcut and arrived at her grandmother’s house first. Because the grandmother thought the wolf was her granddaughter, the wolf was allowed to enter the house and ate the grandmother. A few minutes later, the little girl arrived, and entered her grandmother’s house without realizing that the wolf was in the house, and was also eaten by the wolf. This story is from the fairy tale “Little Red Riding Hood." In order to understand this story, children need to understand the connections between such events as the wolf hurrying to the grandmother’s house and that he arrived there before the little girl, as well as those between such events that the grandmother believed that her granddaughter had come and that she opened the door to allow the wolf to come in. The former connection could be called behavioral connection or causality, and the latter, psychological connection or causality.

Thus, to track sequence of events in the story, children should need to understand behavioral and psychological causality. To understand behavioral causality, knowledge on everyday activities or scripts is needed. To track event sequences connected by psychological causality, children must focus on and track the mental states of protagonists. With these abilities, children may track event sequences in a story. However, those abilities are not enough. To enjoy the twists and turns of 

suspenseful stories, readers are required to compare protagonists’ belief with their own, since protagonists’ perspectives are sometimes different from readers’, and even from the reality (e.g., false belief), which makes readers kept in suspense while reading the story. Actually, the scenes in Little Red Riding Hood become suspenseful when the reader possesses both the character’s perspective (i.e., the Little Red Riding Hood thinks that her grandmother is in the house) and the reader’s own perspective (i.e., the “grandmother” is impersonated by the wolf). To enjoy and be thrilled by a story, readers need the ability to understand protagonists’ false belief, which is different from the readers’ own knowledge of reality. In other words, readers must have the ability to understand others’ false belief, which can be evaluated by using standard false belief tasks (Wimmer and Perner, 1983; Baron-Cohen et al., 1986).

Among the abilities to understand various kinds of causality, understanding psychological causality appeared to be the most closely related to false belief understanding, since both processes require children to attend to and track protagonists’ mental states. In fact, Baron-Cohen et al. (1986) demonstrated that the ability to understand false beliefs and to sequence events based on psychological causality develop concurrently. To do this, they tested children with autism by asking them to sequence pictures to form a story involving psychological causality along with stories with behavioral and mechanical causality. The children succeeded in sequencing pictures based on mechanical or behavioral causality. However, they failed to sequence pictures based on psychological causality. The authors also found a strong correlation between performance in sequencing the psychological pictures and a standard false belief task of the children with autism: 14 out of 19 participants passed or failed both tasks. Thus, Baron-Cohen et al. (1986) suggested that the ability to sequence pictures based on psychological causality is related to the ability to predict protagonists’ subsequent behavior based on their false belief.

Baron-Cohen et al. (1986) did not comment on typically developing children, or the order in which the two examined abilities develop. However, as mentioned above, understanding a protagonist’s false belief requires children to compare Little Red Riding Hood’s false belief with their own knowledge of reality, whereas what children need to connect events based on psychological causality is the ability to attend to and track protagonist’s mental states. Therefore, children would be able to sequence events related to psychological causality (e.g., false belief) successfully before they become able to understand other’s false belief.

The aim of the present study was therefore to investigate the relationship between the ability to sequence picture events including psychological causalities and the ability to understand the protagonist’s false beliefs in a typically developing sample of Japanese children aged 3 to 6. Based on previous research, we felt that this age of children would be well suited for our study. Wellman and Bartsch (1988) indicated that 3-year-old children have begun to show an understanding other’s belief, by demonstrating that 3-year-olds can appropriately predict a character’s actions based on the character’s beliefs. Although whether 3-year-olds can predict a character’s future behavior based on the character’s false belief remains unknown, the development toward understanding of others’ false belief from 3 years is worth investigating. We included 6-year-olds as the oldest participants since previous studies have suggested that Japanese children begin to show successful performance on a standard false belief task around 6 years of age (Wellman et al., 2001; Naito and Koyama, 2006). We predicted that typically developing children would be able to successfully sequence story events that are related by psychological causality (e.g., false beliefs) before 6 years of age when they become able to understand other’s false beliefs (Wellman and Bartsch, 1988; Bartsch and Wellman, 1989).

In the present study, we conducted a longitudinal study and investigated whether children who had successfully sequenced pictures based on psychological causality (TIME 1) would be more likely to pass the standard false belief task 5 months later (TIME 2), compared with those who had failed in the picture-sequencing task at TIME 1. We asked children to sequence pictures not only based on psychological causality, but also based on behavioral as well as mechanical causality. Baron-Cohen et al. (1986) also reported the performance of sequencing pictures of mechanical and behavioral stories in a sample of children with autism; however, that study did not examine whether this performance was related to performance on a standard false belief task. The general ability to follow causal sequences in a story may thus contribute to passing a standard false belief task. We considered that if the general ability to understand causality contributes to children’s passing the standard false belief task, their performance on the false belief task at TIME 2 would not only be related to their ability to sequence psychological story pictures, but also to their ability to sequence mechanical and behavioral pictures at TIME 1. By contrast, if successful sequencing of psychological stories may be an early manifestation of understanding of others’ false beliefs, performance on sequencing pictures in psychological stories at TIME 1 would be most strongly related to performance on the false belief task at TIME 2. Given that children with autism have difficulty understanding causal sequences containing mental states but do not have difficulty understanding general causality (Baron-Cohen et al., 1986), we considered that this prediction was likely to be supported.

In the following sections, we first report children’s performance of three types of picture sequencing tasks and on the task of false belief in Experiment 1. Then, in Experiment 2, we examine whether children who had shown high ability on three types of picture sequencing tasks would perform better in the false belief task at Experiment 2, compared to children with low ability at Experiment 1.

## Experiment 1

### Materials and Methods

#### Participants

Volunteers were recruited from two nursery schools in Fukuoka, Japan. The participants initially included 115 Japanese children; however, five children were excluded from the analysis (one child did not complete the tasks due to fatigue, three children did not respond or answered “I do not know” during some of the tasks, and one child did not understand the Japanese instructions). Thus, the final sample included 110 children: 28 3-year-old children (*M* = 43.11 months, *SD* = 3.71 months, range = 37–47 months; 17 boys, 11 girls), 28 4-year-old children (*M* = 52.86 months, *SD* = 3.46 months, range = 48–58 months; 14 boys, 14 girls), 27 5-year-old children (*M* = 64.78 months, *SD* = 3.43 months, range = 60–70 months; 12 boys, 15 girls), and 27 6-year-old children (*M* = 77.22 months, *SD* = 2.68 months, range = 72–82 months; 16 boys, 11 girls). This study was ethically reviewed and approved by Institutional Review Board for Clinical Research at Osaka University Hospital prior to the study. We adhered to the Declaration of Helsinki and the institutional guidelines for experiments with human participants. Caretakers of all children provided written consent prior to their participation in the study. In addition, at the start of each individual investigation, an experimenter verbally confirmed each participant’s willingness to participate.

#### Materials and Tasks

##### Picture sequencing

**FIGURE 1:**
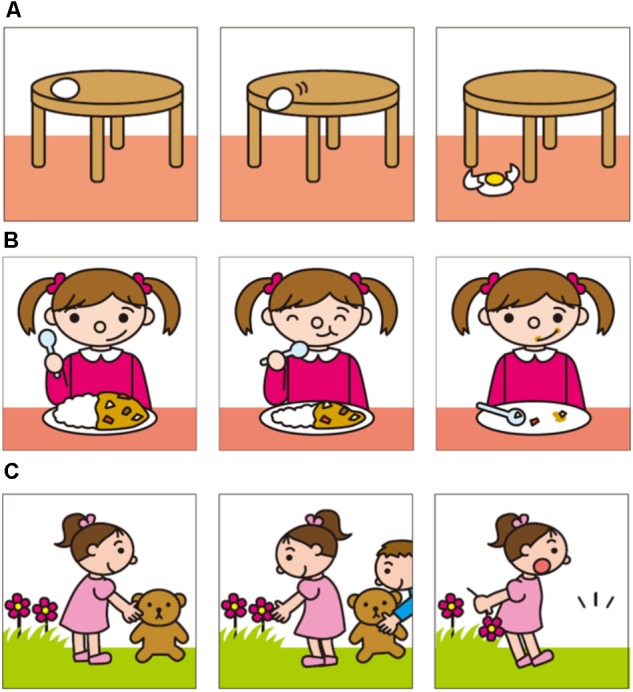
Examples of the picture sequences: **(A)** mechanical causality, **(B)** behavioral causality, and **(C)** psychological causality.

This task assessed whether the participants could arrange pictures in a predetermined sequence. Based on the study by Baron-Cohen et al. (1986), three types of stories were used involving either mechanical, behavioral, or psychological causality. The stories about mechanical causality represented objects interacting causally with each other. The stories about behavioral causality represented one or two person(s) engaging in everyday activities where the sequence could be understood without considering the protagonist’s mental state. The stories about psychological causality represented people acting in everyday activities where tracking the protagonists’ mental states was required by the children in order to grasp the sequence. There were four different stories for each condition (**Table 1**). **Figure 1** provides illustrative examples of the stories used herein. The actual pictures were drawn in color and were displayed on 5 × 5-inch cards. In order to test a younger sample, we chose a three-picture length for each story; this was different from the method used by Baron-Cohen et al. (1986).

**Table 1:** The contents of the four different stories for each condition.

		Picture		
	
		1	2	3
Mechanical causality	1	An egg is on the edge of a table	The egg rolls and is about to fall	The egg falls off the table and breaks
	2	A balloon flies	The balloon flies toward a tree	The balloon bursts on a tree
	3	A rock on a hilltop and a man under the hilltop	The rock rolls down the hill and hits the man	The rock makes the man fall down
	4	A man is under an apple tree	An apple falls toward the man	The apple hits the man on the head
Behavioral causality	1	A girl with a plate full of curry and rice	The girl eats the curry and rice	The girl is with an empty plate
	2	A boy puts on a shirt	The boy puts on shoes	The boy is about to go outside
	3	A boy eats ice cream	A girl is about to take the ice cream	The girl eats the ice cream
	4	A woman takes a miniature car	The woman gives the car to a boy	The boy plays with the car
Psychological causality	1	A girl puts a teddy down	The girl turns away from the teddy to pick a flower and a boy takes the teddy behind her back	The girl is surprised to see the teddy is gone
	2	A girl puts candy in a bag	While the girl is walking, a thief steals the candy from the back	The girl is shocked to find the candy in the bag is gone
	3	A man out a ball into a box	The man turns away from the ball to pick up a doll and a child takes the ball behind his back	The man gets upset to find that the ball is gone
	4	A boy is happy to see cake on the table	The boy turns away to get a fork and his mother eat the cake behind him	The boy has a fork and is surprised to see that the cake is gone

A frame for holding the sequence of the three cards was used in this task; each position was marked with a number. Consistent with previous studies (Baron-Cohen et al., 1986), the experimenter always placed the first of three pictures at the start of the sequence, with the remaining two in random order. Thus, the participant was required to place the remaining two pictures in sequence during each trial. Each participant was given the following instructions: “This is the first picture. Look at the other pictures and see if you can make a story with them.” If a child did not respond, the experimenter asked him or her again as follows: “Which is the next picture?” The experiment proceeded at each participant’s own pace, but participants could only attempt each story once. All participants except five (see information on excluded participants above) completed the task by the second prompt.

##### False belief understanding

Based on Wellman and Liu (2004), an experimenter asked the participants to judge another person’s false belief about what was in a container. In this task, the participant was shown a box of bandages and discovered that it had a miniature horse inside, not bandages. Then, the experimenter showed the participants a puppet and said, “This puppet has not seen inside this box. What does the puppet think is inside the box, bandages or a horse (a horse or bandages; counterbalanced for the half of participants)?”

#### Procedures

Participants were assessed individually in a room in their nursery school. The order of the tasks was counterbalanced across the participants. In addition, the order of the presentation of each picture sequencing trial and the order of the type of picture sequencing were also counterbalanced.

##### Scoring

During the picture-sequencing task, the experimenter recorded the order chosen by each participant. If the participant chose the correct sequencing in a trial, a score of “1” was assigned; if he or she sequenced the pictures in the wrong order, no score was assigned. Thus, the range of scores in each type was between “0” and “4.” Based on the total score, participants were classified into two groups: a low-score group (scores ranging from 0 to 2) and a high-score group (scores of “3” or “4”) in each type.

In the analysis of false belief understanding, participants’ answers were divided into either pass (“bandage”) or fail (“horse”).

### Results

#### Picture Sequencing

**FIGURE 2:**
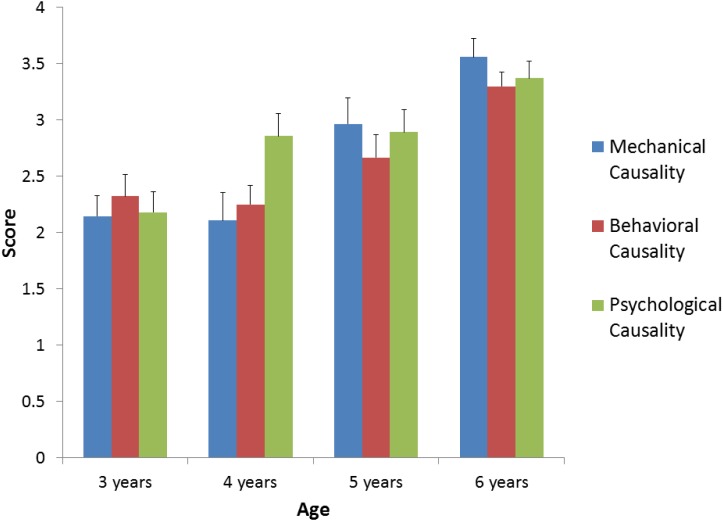
Mean scores of the three conditions (mechanical, behavioral, and psychological causality) in each age group (3-, 4-, 5-, and 6-year-old children).

The mean scores for the three types of stories (mechanical, behavioral, and psychological causality) are shown for each age group (3-, 4-, 5-, and 6-year-old children) in **Figure 2**.

A 3 × 4 analysis of variance was conducted with the types of stories (mechanical, behavioral, or psychological) as the within-subjects factor and the age of participants (3, 4, 5, or 6 years) as the between-subjects factor. The results indicated that there were no significant main effects for type of story, *F*(2,212) = 1.45, *p* = 0.24, but there was a significant main effect for age, *F*(3,106) = 14.90, *p* < 0.001. Scores on the picture sequencing task were higher at 5 years of age than 3 years of age (*p* = 0.01), and higher at 6 years of age than 3, 4, and 5 years of age (*p* < 0.001, *p* < 0.001, and *p* = 0.03 via paired-comparisons with a Bonferroni correction, respectively). There was also a significant interaction between age and type of story, *F*(6,212) = 2.19, *p* = 0.045. Paired comparisons with Bonferroni correction showed that scores on sequencing pictures for stories about mechanical causality increased from 4 to 5 years of age (*p* = 0.03). In addition, scores on sequencing pictures for stories about behavioral causality increased from 4 to 6 years of age (*p* < 0.001). Scores on sequencing pictures about psychological causality stories increased from 3 to 5 years of age (*p* = 0.048). Moreover, 4-year-old children showed better performance in sequencing pictures containing psychological causality when compared to sequencing pictures containing mechanical causality (*p* = 0.005).

The scores for the three types of stories (mechanical, behavioral, and psychological causality) in each age group were compared to the chance level. Given that the experimenter puts the first picture out of three pictures in position, children had a 50% chance of getting each sequence right purely by chance. Accordingly, each score was compared to 50%. For the mechanical causality stories, the performances shown by 5- and 6-year-old children were higher than the chance level, *t*(26) = 4.20, *p* < 0.001; *t*(26) = 9.54, *p* < 0.001, respectively. For the behavioral causality stories, the performances shown by 5- and 6-year-old children were higher than the chance level, *t*(26) = 3.34, *p* = 0.003; *t*(26) = 10.07, *p* < 0.001, respectively. For the psychological causality stories, the performances shown by 4-, 5-, and 6-year-old children were higher than the chance level, *t*(27) = 4.34, *p* < 0.001; *t*(26) = 4.40, *p* < 0.001; *t*(26) = 8.99, *p* < 0.001, respectively. Some knowledge may need for sequencing stories involving mechanical and behavioral causality, but not for sequencing stories involving psychological causality; knowledge about physical rules for mechanical causalities, and knowledge of scripts in daily life for behavioral causalities.

#### False Belief Task

In the false belief task, 43 out of 110 participants answered correctly: 4 out of 28 3-year-olds, 7 out of 28 4-year-olds, 13 out of 27 5-year-olds, and 19 out of 27 6-year-olds. Comparisons against chance indicated that 3- and 4-year-olds’ performance was significantly less than chance (*p* < 0.001 for 3-year-olds and *p* < 0.01 for 4-year-olds via sign tests), 5-year-olds performed at chance, and 6-year-olds performed successfully (*p* < 0.05). Thus, as shown in previous studies (Wellman et al., 2001; Naito and Koyama, 2006), Japanese children begin to show successful performance on a standard false belief task after 6 years of age. Moreover, a comparison of performance across the four age groups indicated that more 6-year-olds passed the false belief task, whereas more 3-year-olds failed the task, χ^2^(3) = 21.60, *p* < 0.01.

#### The Relationship Between Picture Sequencing and False Belief Task

The frequencies of performance on the picture sequencing tasks and the false belief task for each group are presented in **Table 2**. There were significant differences in the proportions of passing the false belief task between the two groups (low or high scores) on the picture sequencing tasks (Fisher’s exact test: mechanical causality: *p* < 0.001; behavioral causality: *p* = 0.002; psychological causality: *p* = 0.003). Regardless of the content of the picture stories, children with low scores on picture sequencing were more likely to fail the false belief task than were children with high scores. Similarly, more children with low scores on the picture-sequencing task failed the false belief task compared to the chance level, whereas children with high scores on the picture-sequencing task passed the false belief task within the chance level. The number of children who received high scores in the picture-sequencing task and failed the false belief task was greater than the number of those who passed the false belief task with low scores on the picture-sequencing task. Thus, the understanding of causality, regardless of the content of the picture stories, preceded the passing of the false belief task.

**Table 2:** The mean age and frequency of children’s performance in the picture sequencing task and the false belief task in Experiment 1.

			False belief task	
	
			Pass	Fail
Picture sequencing	Mechanical causality	Low scores	8 (7.27%) *M* = 55.50 months	40 (36.36%) *M* = 51.78 months
		High scores	35 (31.82%) *M* = 69.40 months	27 (24.55%) *M* = 58.41 months
	Behavioral causality	Low scores	12 (10.91%) *M* = 59.00 months	40 (36.36%) *M* = 52.55 months
		High scores	31 (28.18%) *M* = 69.84 months	27 (24.55%) *M* = 57.26 months
	Psychological causality	Low scores	9 (8.18%) *M* = 67.11 months	34 (30.91%) *M* = 50.65 months
		High scores	34 (30.91%) *M* = 66.74 months	33 (30%) *M* = 58.36 months

The mean age of each group is shown in **Table 2**. The mean age of the participants who failed the false belief task despite having high scores on sequencing pictures was midway between the participants who failed both tasks and those who passed both tasks. An analysis of variance comparing the age of participants among these three groups was significant for mechanical causality, *F*(2,99) = 24.40, *p* < 0.0001, behavioral causality, *F*(2,97) = 21.24, *p* < 0.001, and psychological causality, *F*(2,98) = 15.90, *p* < 0.001. The understanding of mechanical, behavioral, and psychological causality may precede passing the false belief task.

### Discussion

The results of Experiment 1 indicated that the tracking of mechanical, behavioral, and psychological causality was related to understanding other’s false belief. Considering the distribution of children who passed or failed each task, it is possible that tracking causal sequences in mechanical, behavioral, and psychological stories can be achieved earlier than understanding other’s false belief. Specifically, when children could not sequence the pictures in each story, they were more likely to fail the false belief task. However, when they could sequence the pictures of each story, the possibility of passing the false belief task was at chance level. Thus, whether the ability to track causality among pictures directly guides children to understand others’ false belief is still unclear. To find an answer to this question, we conducted Experiment 2 by testing children who failed to pass the false belief task in Experiment 1 5 months later. In Experiment 2, we examined whether the children would become able to succeed in the false belief task and saw the relationship between the performance on picture sequencing task in Experiment 1 and that on the false belief task in Experiment 2.

If children’s performances on the false belief task in Experiment 2 is related not only to their ability to sequence psychological story pictures but also to their ability to sequence mechanical and behavioral pictures in Experiment 1, we could conclude that the general ability to understand causality contributes to children’s understanding of other’s false belief. By contrast, if passing the false belief task in Experiment 2 is related to the performance on neither the mechanical picture sequencing nor the behavioral picture sequencing, but to the performance on psychological picture sequencing, we could regard the successful sequencing of psychological stories as an early manifestation of the ability to understand other’s false belief. Therefore, Experiment 2 investigated whether the children’s performance on picture sequencing tasks found in Experiment 1 would be related to the performance on the false belief task assessed 5 months later.

## Experiment 2

### Materials and Methods

#### Participants

We attempted to contact the 67 participants who failed the false belief task in Experiment 1; however, 14 participants were not contacted because they were no longer enrolled in either of the participating nursery schools. Three children were also excluded from the analysis (two children did not respond, and one child was not present due to hospitalization). The final sample included 50 children. The children were distributed as follows: 35 children with low scores and 15 children with high scores on the sequencing of the mechanical pictures in Experiment 1; 31 children with low scores and 19 children with high scores on the sequencing of the behavioral pictures in Experiment 1; and 28 children with low scores and 22 children with high scores on the sequencing of the psychological pictures in Experiment 1.

#### Materials and Tasks

All participants completed a false belief task (Wellman and Liu, 2004) identical to the task conducted in Experiment 1.

#### Procedures

Experiment 2 was conducted 5 months after Experiment 1; identical procedures were followed in Experiment 2 as in Experiment 1.

### Results

The frequencies of each group’s performance on the two tasks are displayed in **Table 3**. For the sequencing of the psychological pictures, there was a significant difference between the two groups (low or high scores) in the proportion of passing the false belief task (Fisher’s exact test: *p* < 0.001). In order to examine whether this effect was mediated by age, a 2 × 2 analysis of variance was conducted with group (high or low) for the sequencing of the psychological causality pictures and the group (pass or fail) for the false belief task as the between-subjects factors. The results indicated that there were no main effects of picture sequencing, *F*(1,46) = 1.85, *p* = 0.18, no main effects of false belief task, *F*(1,46) = 2.02, *p* = 0.16, and no significant interaction between the two factors, *F*(1,46) = 0.31, *p* = 0.58.

**Table 3:** The mean age and frequency of children’s performance in the picture sequencing task in Experiment 1 and the false belief task in Experiment 2.

			False belief task (Experiment 2)	
	
			Pass	Fail
Picture sequencing (Experiment 1)	Mechanical causality	Low scores	8 (16%) *M* = 50.83 months	27 (54%) *M* = 49.35 months


		High scores	7 (14%) *M* = 62.43 months	8 (16%) *M* = 50.50 months
	Behavioral causality	Low scores	9 (18%) *M* = 58.67 months	22 (44%) *M* = 49.36 months
		High scores	6 (12%) *M* = 56.33 months	13 (26%) *M* = 49.08 months
	Psychological causality	Low scores	5 (10%) *M* = 53.60 months	23 (46%) *M* = 48.11 months
		High scores	13 (26%) *M* = 56.00 months	9 (18%) *M* = 53.78 months

In addition, there were no significant differences in the proportion of passing the false belief task between the two groups (low or high scores) for the sequencing of the mechanical or behavioral pictures (Fisher’s exact test: *p* = 0.11 and *p* = 1.0, respectively).

### Discussion

The children who received high scores on sequencing the psychological pictures in Experiment 1 were more likely to pass the false belief task in Experiment 2 than those who had low scores. This effect was not mediated by age, because those children who showed understanding of other’s false belief in Experiment 2 were as old as those who did not show it.

In contrast, the understanding of mechanical and behavioral causality in Experiment 1 was not related to performance on the false belief task in Experiment 2. The longitudinal findings indicated that the general understanding of causal sequences did not affect the understanding of false beliefs.

## General Discussion

The present study was a longitudinal study that investigated whether children who had successfully sequenced pictures based on mechanical, behavioral, and psychological causality (Experiment 1) would be more likely to understand other’s false belief 5 months later (Experiment 2), compared with those who had failed in the picture-sequencing task. The results from these two experiments suggested that only understanding of psychological causality rather than mechanical or behavioral causality predicts false belief understanding. Thus, typically developing children understand psychological causality in a story before they show an understanding of other’s false beliefs, thereby indicating that children who can sequence pictures by attending to and tracking the protagonists’ mental states would be soon able to understand other’s false belief. On one hand, sequencing pictures based on psychological causality requires children to attend to and track protagonists’ mental states. On the other hand, to pass the false belief task, children need to simultaneously represent others’ false belief and their own knowledge on reality, which should enable children to enjoy the twists and turns of suspenseful stories.

The results demonstrated that the ability to correctly sequence pictures about psychological stories emerges earlier than passing the false belief task. This order of emergence can be explained from two points of view. First, false belief tasks require the ability to predict another’s behavior based on her false belief (Wimmer and Perner, 1983; Wellman et al., 2001), but picture sequencing tasks do not require this ability. Picture sequencing tasks only require the ability to arrange several pictures according to psychological causality. In this task, the behavior that results from the protagonist’s false belief is provided as one of the pictures to be arranged. Bartsch and Wellman (1989) also found that children who failed to correctly predict a protagonist’s behavior based on his/her false beliefs via a standard false belief task were able to explain the protagonist’s behavior with references to the protagonist’s false beliefs. Therefore, a child should be able to explain the reason for a behavior via false beliefs more easily than predicting a behavior via false beliefs. In this respect, a picture sequencing method may be useful in the assessment of young children’s ability to gain theory of mind.

Second, in standard false belief tasks, although children know what is in the box of bandages, they need to ignore their own knowledge and only consider the protagonist’s false mental representation. In contrast, they do not have to do this when sequencing psychological pictures. This explanation is consistent with the response account proposed by Scott and Baillargeon (2009). Specifically, they discussed the phenomenon that children do not pass standard false belief tasks until age four, yet infants younger than 2 years of age show sensitivity to false beliefs when tested via violation-of-expectation methods (Onishi and Baillargeon, 2005; Southgate et al., 2007; Surian et al., 2007; Buttelmann et al., 2009). According to Scott and Baillargeon (2009), at least three processes are involved in passing false belief tasks: a false belief representation process, a response-selection process, and a response-inhibition process (Scott and Baillargeon, 2009; Baillargeon et al., 2010). A false belief representation process requires children to represent the agent’s false belief; however, a response-selection process requires children to access their representation of the agent’s false belief in order to respond. Finally, a response-inhibition process requires children to inhibit answering the test based on their own knowledge (Roth and Leslie, 1998; Birch and Bloom, 2003). However, the number of processes required to respond in the picture sequencing task is fewer than in the false belief task. That is, standard false belief tasks require all three processes, whereas the picture sequencing task requires two processes (i.e., a false-belief-representation process and a response-selection process).

The response account is compelling because it explains why children who do not pass standard false belief tasks can successfully arrange pictures of psychological causality. It also explains why children who have sensitivity to others’ false beliefs from infancy need time to successfully apply this ability to the sequencing of psychological pictures. Indeed, it is not until early childhood that the response-selection process begins to operate smoothly (Obhi and Haggard, 2004; Lebel et al., 2008; Baillargeon et al., 2010). Furthermore, sequencing pictures along a time course also requires children be sensitive to temporal aspects of an event. However, various lines of research have suggested that it is not until 4 years of age that children begin to show this ability (Nelson, 1992; Miyazaki and Hiraki, 2006). These findings suggest that deciding the order of picture cards is difficult for children before the age of four.

In summary, the findings of the present study indicated that children who cannot understand other’s false belief are able to understand and enjoy stories containing false beliefs. Certainly, the children who participated in the current study were only required to arrange three pictures comprising one episode, but they appear to enjoy stories consisting of multiple episodes in everyday picture-book reading situations. In fact, Poulsen et al. (1979) reported that 4-year-old children could infer and attribute mental states to the characters in a picture story consisting of 15 to 18 scenes. Thus, if pictures are arranged as a story, young children who cannot arrange pictures by themselves are still able to understand and enjoy stories that require them to infer psychological causality. This suggests that children who cannot understand other’s false belief may be able to track the causal sequences in a story, such as those depicted in “Little Red Riding Hood.” Indeed, young children may develop the ability to think about the temporal aspects of an event, to infer mental states to bridge episodes, and to use mental states to predict a protagonist’s future behavior while being read a story. Further research may consider examining the relationship between the early experiences of listening to stories, the understanding of psychological sequences, and the prediction of others’ behavior based on their false beliefs; this line of study would be useful in establishing optimal environments for children’s social cognitive development.

## Author Contributions

WS and EH designed the study, read the draft and approved it. WS collected and analyzed the data, and drafted the manuscript.

## Conflict of Interest Statement

The authors declare that the research was conducted in the absence of any commercial or financial relationships that could be construed as a potential conflict of interest.
